# Impact of Early Glycemic Variability on Mortality and Neurologic Outcome of Very Low Birth Weight Infants: Data from a Continuous Glucose Monitoring System*

**DOI:** 10.34763/devperiodmed.20192301.0714

**Published:** 2019-04-08

**Authors:** Mateusz Jagła, Izabela Szymońska, Katarzyna Starzec, Przemko Kwinta

**Affiliations:** 1 Chair of Pediatrics, Jagiellonian University Medical College, Krakow, Poland

**Keywords:** hyperglycemia, hypoglycemia, glycemic variability, continuous glucose monitoring, very low birth weight, hiperglikemia, hipoglikemia, zmienność glikemii, ciągłe monitorowanie glukozy, bardzo mała masa ciała

## Abstract

**Background:**

Glycemic variability (GV) has been a matter of interest in recent years. However, glycemic variability in preterm infants has not been adequately investigated.

**Objectives:**

To evaluate the impact of glycemic variability obtained from continuous glucose monitoring on mortality and neurologie outcomes: grade 3 or 4 intraventricular hemorrhage (IVH), periventricular leukomalacia (PVL), and retinopathy of prematurity (ROP) requiring treatment among very low birth weight infants.

**Material and methods:**

A prospective, single-center, open cohort study enrolled 74 very low birth weight infants with a mean birthweight of 1066 g (+l-267). A continuous glucose monitoring system (CGM) was used to measure glucose during the first week of life. The impact of glycemic variability (standard deviation SD; coefficient of variation CV; and mean amplitude of glucose excursion MAGE) on mortality and neurologie outcomes of infants was evaluated.

**Results:**

Univariate analysis revea/ed that glycemic variability occurring during the first week of life was not be associated with mortality before term-equivalent age and PVL. Higher GV was associated with grade 3 or 4 IVH (CV p=0.025; MAGE p=0.032) and ROP requiring treatment (SD p=0.019; CV p=0.026; MAGE=0.029). However, logistic regression models did not show a significant association between GV occurring during the first week of life and grade 3 or 4 IVH (MAGE OR 2.64; 95% Cl 0.71-9.92) or ROP requiring treatment (MAGE OR 1.14; 95% Cl 0.57-5.32).

**Conclusions:**

Further prospective studies are needed to fully investigate the impact of GV on mortality and morbidity in premature infants. The potential benefits of reducing glucose blood fluctuations in VLBW infants need to be addressed.

## Streszczenie

**Wstęp:**

Zmienność glikemii (GV) jest przedmiotem badań w ostatnich latach. Nie oceniano znaczenia zmienności glikemii u noworodków urodzonych przedwcześnie.

**Cel:**

Ocena wpływu zmienności glikemii uzyskanej z ciągłego monitorowania glikemii na śmiertelność i powikłania neurologiczne: krwawienie dokomorowe stopnia 3 lub 4 (IVH), leukomalację okołokomorową (PVL) i retinopatię wcześniaków (ROP) wymagającą leczenia u noworodków z bardzo małą urodzeniową masą ciała.

**Materiał i metody:**

Prospektywne, jednoośrodkowe, otwarte badanie kohortowe obejmujące 74 noworodki o bardzo małej masie urodzeniowej (1066+/-267 g). Do pomiaru glukozy w pierwszym tygodniu życia stosowano system ciągłego monitorowania (CGM). Oceniano wpływ zmienności glikemii (odchylenia standardowego, SD; współczynnika zmienności, CV; oraz średnią amplitudę odchyleń glikemii, MAGE) na śmiertelność i powikłania neurologiczne.

**Wyniki:**

Analiza jednoczynnikowa wykazała, że zmienność glikemii występująca w pierwszym tygodniu życia nie była związana ze śmiertelnością i PVL. Wyższa GV korelowała z IVH stopnia 3/4 (CV p=0,025; MAGE p=0,032) i wymagającą leczenia ROP (SD p=0,019; CV p=0,026, MAGE = 0,029). Jednakże, w wieloczynnikowej regresji logistycznej nie wykazano związku między GV a IVH stopnia 3/4 (MAGE OR 2,64; 95% Cl 0,71-9.92) lub wymagającego leczenia ROP (MAGE OR 1,74; 95% Cl 0,57-5,32).

**Wnioski:**

Konieczne są dalsze prospektywne badania w celu pełnego zbadania wpływu GV na śmiertelność i zachorowalność u noworodków przedwcześnie urodzonych. Należy rozważyć potencjalne korzyści z ograniczenia wahań stężeń glukozy u noworodków z bardzo małą masą urodzeniową.

## Introduction

Glucose concentration depends on large number of factors and is characterized by constant fluctuations. Therefore, the full spectrum of glucose disturbance involves not only hypoglycemia and hyperglycemia but also glycemic variability (GV). It has been shown that increased glycemic variability is associated with oxidative stress, which results in direct cellular damage and apoptosis [1]. When adults in intensive care were investigated, high GV was an independent risk factor for mortality[2]. The relationship between GV and mortality was also reported in very low-birthweight (VLBW) infants and term neonates, but continuous glucose monitoring was not always used [3, 4]. The present study evaluates the possible association between GV during the first week of life and mortality and neurological morbidity among VLBW infants. Glucose measurements were obtained prospectively from a continuous glucose monitoring system (CGM) system.

## Aim

To evaluate the impact of early glycemic variability on mortality and neurologic outcomes: grade 3 or 4 intraventricular hemorrhage (IVH), periventricular leukomalacia (PVL), and treatment-requiring retinopathy of prematurity (ROP) among very low birth weight infants.

## Patients and methods

### Patients

From January 2013 to January 2015, a single-center prospective study was conducted at the Neonatal Intensive Care Unit of the Department of Pediatrics, Jagiellonian University, a third level neonatal center in Krakow, Poland. Neonates were eligible for the study if they weighed 1500 g or less at birth and were admitted to the unit during the first day of life. Exclusion criteria were: major congenital malformations, a diabetic mother, a suspicion of an inborn error of metabolism, perinatal trauma, and asphyxia.

### Glucose measurements

A continuous glucose monitoring system was used to measure glucose (Guardian Real-Time CGM®, Medtronic, Northridge, USA). The continuous glucose monitoring sensor (SofSensor®, Medtronic, Northridge, USA) was inserted into the subcutaneous tissue of the lateral side of the thigh, from which measurements were recorded every five minutes for six days. Both the accuracy and safety of the CGM system have been validated in VLBW neonates [5]. The CGM system was calibrated at least three times a day with a blood sample obtained from the radial or umbilical arterial lines (which were infused only with a normal saline solution). Point of care blood glucose analysis of samples routinely used for clinical management (Cobas b 221 POC, Roche Diagnostics, Germany) were used to calibrate the CGM system. The normal range of interstitial glucose concentration was predefined between 70 and 180 mg/dL. The incidence of hyperglycemia and hypoglycemia based on sensor interstitial glucose concentration was investigated. Glycemic variability (GV) was assessed by standard deviation (SD), coefficient of variation (CV) calculated as the percent value of SD divided by the mean glucose and mean amplitude of glucose excursion (MAGE) defined as

**Figure:**
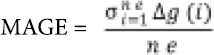


where *ne* is the number of excursions with amplitudes greater than one SD and *Ag( i)* is the *i*th glucose excursion greater than the SD of the whole monitoring. Both the data from the CGM system as well as the calibration values were not blinded and were available for clinicians. A uniform nutrition strategy and intervention protocol was used in the cases of predefined hyper- or hypoglycemia.

### Outcomes

The objective was to evaluate the ability of early glycemic variability to predict death (primary outcome) and neurologic morbidity (secondary outcomes): retinopathy of prematurity requiring treatment (ROP), severe intraventricular hemorrhage (grade 3 or 4 IVH), and periventricular leukomalacia (PVL). We selected the following biomarkers of interest: glycemic variability assessed by the standard deviation of the mean glycemia (SD), the coefficient of variation (CV) and the mean amplitude of glycemic excursion (MAGE), episodes of hypoglycemia (<70 mg/dL), and episodes of hyperglycemia (>150 mg/dL).

The diagnosis of intraventricular hemorrhage was determined through cranial ultrasonography (Envisor HD 11, Philips, USA) performed, according to the standardized protocol, within the first day of life, then at 1 week of life, and subsequently at 1- to 2-week intervals. Hemorrhage grades 1 and 2 were determined as mild hemorrhage and grades 3 and 4 as severe. All ultrasonography was performed by neonatologists certified by the Polish Society of Ultrasonography.

Periventricular leukomalacia was recognized by magnetic resonance imaging of the brain performed for infants at near term-equivalent age (GA of 36-39 weeks) by using a 1.5 T (General Electric Healthcare, Milwaukee, USA) magnetic field induction MR system with 8-channel coil. Morphological brain changes were assessed using standard T1-weighted, T2-weighted and T2-FLAIR sequences. The scans were analyzed by a single neuroradiologist experienced in pediatric MRI examination.

Retinopathy of prematurity was diagnosed according to the guidelines of the International Classification of Retinopathy of Prematurity revisited [6]. Stages of ROP were determined according to the International Classification of ROP (Stages 1-5). In this study, examinations were performed by a pediatric ophthalmologist after mydriasis using a binocular indirect ophthalmoscope (Keeler Instruments Inc., USA) and was verified by digital imaging using a RetCam imaging system (Clarity Medical Systems, USA). Laser therapy was performed in infants with indications outlined in the ETROP criteria (ROP 1 Type): any stage of ROP with plus disease in Zone I; stage 3 ROP without plus disease in Zone I; stage 2 or 3 ROP with plus disease in Zone II.

### Ethics

The study protocol was approved by the Jagiellonian University Ethics Committee, and written informed consent was obtained from a parent of each infant.

### Statistical analysis

Categorical variables are presented as numbers (n) and percentages (%); continuous variables are presented as median (interquartile range, IQR or 95% CI for median), arithmetic mean (± standard deviation, SD) for normally distributed data, and geometric mean (÷/× geometric standard deviation, GSD) for non-normal positively skewed data.

First, an initial univariate analysis was performed to determine the risk factors associated with outcomes, utilizing either Student's two-tailed t-test, Welch’s test, or the Mann-Whitney test to compare continuous variables and Fisher’s exact test to compare categorical values. Then, multiple logistic regression was used to evaluate the risk factors determined by univariate analyses. The odds ratio (OR) and 95% confidence interval (CI) were calculated for each risk factor. Assumptions of linearity and normally distributed residuals were visually checked using residual and P-P plots. The variance inflation factor (VIF) was used to exclude multicollinearity among predictors in the multiple logistic regression model. Probability values (p-values) below 0.05 were considered statistically significant. Analysis was carried out using MedCalc Statistical Software (version 16.8.4, Ostend, Belgium; https://www.medcalc.org; 2016).

## Results

### Patient characteristics

Seventy-four patients were enrolled in the study. The baseline demographic and clinical characteristics of the patients are detailed in table I.

**Table I:** Demographic and clinical characteristics of the patients. Tabela I. Charakterystyka kliniczna i demograficzna pacjentów.

Number of patients *Liczba pacjentów*	74
Birth weight, g, mean (SD) *Masa urodzeniowa, g, średnia (SD)*	1066 (267)
Gender, male n (%) *Płeć męska, n (%)*	44 (59.5)
GA, wk median (IQR) *Wiek płodowy, tyg., mediana (IQR)*	28 (26-30)
CRIB II score, median (IQR) *Skala CRIB II, mediana (IQR)*	7(5-10)
Small for GA, n (%) *Mały do wieku płodowego, n (%)*	6(8.1)
Antenatal steroids, n (%) *Steroidy prenatalnie, n (%)*	47 (62.6)
Death before 7 DOL, n (%) *Zgon przed* 7 *dobą życia, n (%)*	3 (4.0)
Death before term-equivalent age, n (%) *Zgon przed osiągnięciem wieku skorygowanego, n (%)*	7(9.5)

SD–standard deviation, IQR – interquartile range, GA–gestational age, CRIB II – Clinical Risk Index for Babies II, DOL–day of life SD – odchylenie standardowe, IQR – rozstęp kwantylowy, GA – wiek płodowy, CRIB II – skala CRIB, DOL – doba życia

### Interstitial fluid glucose concentration and glycemic variability

A total of 102,334 glucose measurements were delivered by the continuous glucose monitoring system. The average time below 70 mg/dL was 3.14%, and above 150 mg/dL, 10.35%. The mean glucose concentration and ranges of selected methods of glycemic variability assessment were calculated and are presented in table II. This shows the arithmetic means and standard deviation (SO) or the geometric means and geometric standard deviation (GSD) for positively skewed data.

**Table II:** Interstitial fluid glucose concentration and glycemic variability indices in the cohort of very low birth weight infants (n=74). Tabela II. Śródtkankowe stężenie glukozy i wskaźniki zmienności glikemii w kohorcie noworodków z bardzo małą masą ciała (n=74).

Parameter *Parametr*	Value *Wartość*
Glucose, mean (*-/*+SD) *Glukoza, średnia (-/+SD)*	5.96 (0.71)
mean, SD (7+SD) *średnia, SD (-/+SD)*	1.24 (0.37)
CV, geometric mean (+/^×^GSD) *CV, średnia geometryczna (÷/×GSD)*	21.24 (5.58)
MAGE, geometric mean (*÷*/^×^GSD) *MAGE, średnia geometryczna (÷/×GSD)*	1.89 (1.34)

Glucose was measured in mmol/L. Glycemic variability indices: SD – Standard Deviation of the Mean Glucose; CV – glucose Coefficient of Variation; MAGE – Mean Amplitude of Glycemic Excursion SD – standard deviation; GSD – geometric standard deviation *Stężenie glukozy wyrażono w mmol/l*. Wskaźniki zmienności glikemii: SD – Odchylenie Standardowe od średniej glukozy; CV– Współczynnik Zmienności glukozy; MAGE– Średnia Amplituda Odchyleń Glukozy; SD – odchylenie standardowe, GSD – geometryczne odchylenie standardowe

### Mortality

Univariate analysis revealed that glycemic variability occurring in the first week of life, glucose median concentration, incidence of hypoglycemia <70 mg/dL, and incidence of hyperglycemia >150 mg/dL were not be associated with mortality before term-equivalent age (tab. III).

**Table III:** Univariate analysis of glucose disturbances associated with death, n=74. Tabela III. Jednoczynnikowa analiza zależności zaburzeń glikemii i zgonu, n=74.

	Death *Zgony* n=7	Survivors *Przeżycie* n=67	p value *wartość p*
Glucose, median (95% Cl for median) *Glukoza, mediana (95% Cl dla mediany)*	6.01 (5.37-8.48)	5.95 (5.67-6.19)	0.548^mw^
Hypoglycemia <70 mg/dL, median (95% Cl for median) *Hipoglikemia <70 mg/dL, mediana (95% Cl dla mediany)*	75 (22-172)	32 (22-44)	0.202^mw^
Hyperglycemia >150 mg/dL, median (95% Cl for median) *Hiperglikemia 150 mg/dL, mediana (95% Cl dla mediany)*	119 (0.0-508)	47 (20-112)	0.664^mw^
SD of glucose (95% Cl) *SD glukozy (95% Cl)*	1.68 (0.97-2.93)	1.23 (1.14-1.34)	0.226^w^
CV of glucose (95% Cl) *CV glukozy (95% Cl)*	26.15 (17.31-39.51)	20.43 (19.28-21.82)	0.205^w^
MAGE (95% Cl) *MAGE (95% Cl)*	2.28 (1.49-3.47)	1.87 (1.75-1.99)	0.295^”^

t - two tailed t Student test after log transformation; w – two tailed t Student test after log transformation with a correction for unequal variances (Welch test); mw– Mann–Whitney test; p<0.05 indicates statistical significance; substandard Deviation of the Mean Glucose; CV–glucose Coefficient of Variation; MAGE – Mean Amplitude of Glycemic Excursion; Cl – confidence interval t – dwustronny test t– Studenta po przekształceniu logarytmicznym; t – dwustronny test t– Studenta po przekształceniu logarytmicznym z korekcją na nierówne wariancje (test Welcha), mw – test Whitney'a i Manna; p<0.05 – poziom istotności; SD – odchylenie standardowe średniego stężenia glukozy; CV – współczynnik zmienności glukozy; MAGE – średnia amplituda odchyleń glukozy; Cl – przedział ufności

### Neurologic outcomes

Univariate analysis suggested an increased risk for development of severe intraventricular hemorrhages for CV (p=0.025) and MAGE (p=0.032) (tab. IV). Simple analysis also revealed an increased risk for ROP requiring treatment for hyperglycemia (p=0.018) and glycemic variability (SD p=0.019; CV p=0.026; MAGE=0.029) (tab. V). There was no association between glucose disturbances occurring on the first week of life and risk for PVL (tab. VI).

**Table IV:** Univariate analysis of glucose disturbances associated with grade 3 or 4 IVH (severe IVH), n=74. Tabela IV. Jednoczynnikowa analiza zależności zaburzeń glikemii i krwawienia dokomorowego stopnia 3 lub 4, n=74.

	Severe IVH *Ciężki IVH* n=ll	Non–severe IVH *Brak ciężkiego IVH* n=63	p value
Glucose, median (95% Cl for median) *Glukoza, mediana (95% Cl dla mediany)*	5.92 (5.45-7.24)	5.99 (5.68-6.24)	0.885^mw^
Hypoglycemia <70 mg/dL, median (95% Cl for median) *Hipoglikemia <70 mg/dL, mediana (95% Cl dla mediany)*	37 (23.28-123.60)	32 (21.25-49.24)	0.273^mw^
Hyperglycemia>150 mg/dL, median (95% Cl for median) *Hiperglikemia 150 mg/dL, mediana (95% Cl dla mediany)*	47 (17-341)	49 (17-118)	0.697^mw^
SD of glucose (95% Cl) *SD glukozy (95% Cl)*	1.55 (1.12-2.16)	1.23 (1.13-1.34)	0.052^t^
CV of glucose (95% Cl) *CV glukozy (95% Cl)*	25.03 (19.68-31.83)	20.32 (19.02-21.72)	0.025^t^
MAGE (95% Cl) *MAGE (95% Cl)*	2.27 (1.79-2.88)	1.85 (1.73-1.98)	0.032^*^

t – two tailed t Student test after log transformation; w – two tailed t Student test after log transformation with a correction for unequal variances (Welch test); mw – Mann–Whitney test; p<0.05 indicates statistical significance; SD–Standard Deviation of the Mean Glucose; CV–glucose Coefficient of Variation; MAGE – Mean Amplitude of Glycemic Excursion; Cl – confidence interval t – dwustronny test t– Studenta po przekształceniu logarytmicznym; t – dwustronny test t – Studenta po przekształceniu logarytmicznym z korekcją na nierówne wariancje (test Welcha), mw – test Whitney'a i Manna; p<0.05 – poziom istotności; SD – odchylenie standardowe średniego stężenia glukozy; CV– współczynnik zmienności glukozy; MAGE – średnia amplituda odchyleń glukozy; Cl – przedział ufności

**Table V:** Univariate analysis of glucose disturbances associated with ROP requiring treatment, n=67. Tabela V. Jednoczynnikowa analiza zależności zaburzeń glikemii i ROP wymagającej leczenia, n=67.

	ROP n=22	Non – ROP n=45	P value
Glucose, median (95% Cl for median) *Glukoza, mediana (95% Cl dla mediany)*	6.15 (5.96-6.68)	5.81 (5.49-6.10)	0.071^mw^
Hypoglycemia <70 mg/dL, median (95% Cl for median) *Hipoglikemia <70 mg/dL, mediana (95% Cl dla mediany)*	32 (11.95-75.28)	32 (20.46=54.23)	0.898^mw^
Hyperglycemia >150 mg/dL, median (95% Cl for median) *Hiperglikemia 150 mg/dL, mediana (95% Cl dla mediany)*	131 (58.81-266.9)	24 (10.92-71.11)	0.018^mw^
SD of glucose (95% Cl) *SD glukozy (95% Cl)*	1.42 (1.22-1.66)	1.16 (1.06-1.27)	0.019^t^
CV of glucose (95% Cl) *CV glukozy (95% Cl)*	22.73 (20.06-25.76)	19.57 (18.23-21.02)	0.026^t^
MAGE (95% Cl) *MAGE (95% Cl)*	2.08 (1.87-2.33)	1.79 (1.65-1.94)	0.029^t^

t- two tailed t Student test after log transformation; w– two tailed t Student test after log transformation with a correction for unequal variances (Welch test); mw– Mann-Whitney test; p<0.05 indicates statistical significance; SD–Standard Deviation of the Mean Glucose; CV – glucose Coefficient of Variation; MAGE – Mean Amplitude of Glycemic Excursion; Cl – confidence interval t– dwustronny test t– Studenta po przekształceniu logarytmicznym; t – dwustronny test t– Studenta po przekształceniu logarytmicznym z korekcją na nierówne wariancje (test Welcha), mw – test Whitney'a i Manna; p<0.05 – poziom istotności; SD – odchylenie standardowe średniego stężenia glukozy; CV – współczynnik zmienności glukozy; MAGE – średnia amplituda odchyleń glukozy; Cl – przedział ufności

**Table VI:** Univariate analysis of glucose disturbances associated with PVL, n=67. Tabela VI. Jednoczynnikowa analiza zależności zaburzeń glikemii i PVL, n=67.

	PVL n=6	Non – PVL n=61	p value
Glucose, median (95% Cl for median) *Glukoza, mediana (95% Cl dla mediany)*	6.55 (5.37-7.06)	5.95 (5.67-6.11)	0.323^t^
Hypoglycemia <70 mg/dL, median (95% Cl for median) *Hipoglikemia <70 mg/dL, mediana (95% Cl dla mediany)*	39.0 (1-107)	32.0 (21-45)	0.947^mw^
Hyperglycemia >150 mg/dL, median (95% Cl for median) *Hiperglikemia 150 mg/dL, mediana (95% Cl dla mediany)*	158.5 (14-306)	47 (17-109)	0.286^mw^
SD of glucose (95% Cl) *SD glukozy (95% Cl)*	1.42 (1.01-1.98)	1.23 (1.12-1.33)	0.314^t^
CV of glucose (95% Cl) *CV glukozy (95% Cl)*	22.54 (17.70-28.69)	20.37 (19.05-21.79)	0.369^t^
MAGE (95% Cl) *MAGE (95% Cl)*	2.34 (1.81-3.03)	1.84 (1.72-1.97)	0.051^t^

t– two tailed t Student test after log transformation; w- two tailed t Student test after log transformation with a correction for unequal variances (Welch test); mw – Mann-Whitney test; p<0.05 indicates statistical significance; SD–Standard Deviation of the Mean Glucose; CV – glucose Coefficient of Variation; MAGE – Mean Amplitude of Glycemic Excursion; Cl – confidence interval t– dwustronny tests– Studenta po przekształceniu logarytmicznym; t – dwustronny test t– Studenta po przekształceniu logarytmicznym z korekcją na nierówne wariancje (test Welcha), mw – test Whitney'a i Manna; p<0.05 – poziom istotności; SD – odchylenie standardowe średniego stężenia glukozy; CV – współczynnik zmienności glukozy; MAGE – średnia amplituda odchyleń glukozy; Cl – przedział ufności

Multivariate linear regression was performed to examine the associations between severe and explanatory variables selected based on results of univariate analyses and earlier research. To reduce multicollinearity SD and CV were removed from multiple regression model. The multivariate linear regression model revealed that only gestational age (OR 0.98; 95% CI 0.97–0.99) and duration of oxygen exposure (OR 1.02, 95% CI 1.01-1.36) are associated with severe ROP. The multivariate model did not show a statistical association between MAGE and risk for ROP requiring treatment (OR 1.74; 95% CI 0.57-5.32). The Hosmer-Lemeshow test result indicated the fitness of the overall model (tab. VII).

Multivariate analysis did not show a statistical association between glycemic variability and risk for severe intraventricular hemorrhages (OR 1.31; 95% CI 0.16-10.67). No interaction or multicollinearity problems were observed for the final model. The Hosmer-Lemeshow test result was 0.129, which indicated the fitness of the overall model (tab. VII).

**Table VII:** Multivariate linear regression model. Tabela VII. Wieloczynnikowa regresja logistyczna.

*Zmienne* Variables *modelu*	B	Wald X^2^	P	OR (95% Cl)
ROP requiring treatment *ROP wymagająca leczenia* Gestation age *Wiek płodowy* Oxygen exposure *Narażenie na tlen* Hyperglycemia *Hiperglikemia* MAGE	-0.01 0.07 -0.01 2.64	6.53 4.68 4.93 8.29	0.011 0.031 0.06 0.149	0.98 (0.97-0.99) 1.02 (1.01-1.36) 0.98 (0.99-1.01) 1.74 (0.57-5.32)
Hosmer-Lemeshow test 0.179				
Severe IVH *Ciężkie IVH* Gestation age *Wiek płodowy* Antenatal GCS *Steroidy prenatalnie* MAGE	-0.54 -0.93 0.56	6.11 0.88 0.95	0.04 0.29 0.33	(0.38-0.87) 0.39 (0.06-2.22) 1.31 (0.16-10.67)
Hosmer-Lemeshow test 0.127				

Birth weight, SD and CV of glucose were removed from the model to exclude multicollinearity (VIF>2). Odds ratio (OR) for each: week of oxygen exposure FiO2 > 0.21; week of gestation age; each episode of hyperglycemia > 150 mg/dL Severe IVH – intraventricular haemorrhage grade 3 or 4 Masa urodzeniowa, SD i CV zostały usunięte z modelu z powodu współliniowości mnogiej (VIF>2). Iloraz szans (OR) dla każdego: tygodnia ekspozycji na tlen FiO2> 0.21; tygodnia wieku ciążowego; każdego epizodu hiperglikemii > 150 mg/dl Ciężki IVH – krwawienie dokomorowe stopnia 3 lub 4

## Discussion

Our study was the first time, to our knowledge, that the impact of early glycemic variability on mortality and neurologic outcome of very low birth weight infants was investigated.

There is very limited data for glycemic variability in premature infants and its role in pathogenesis of diseases connect with prematurity. Both hypoglycemia and hyperglycemia are common findings among preterm infants and both have been associated with increased mortality and morbidity [7, 8]. Hyperglycemia can increase risk of intraventricular hemorrhage, patent ductus arteriosus and retinopathy of prematurity [9, 10]. Hypoglycemia, on the other hand, is connected with characteristic occipital lesions [11]. Rapid blood glucose fluctuations had an even more specific triggering effect on oxidative stress than chronic sustained hyperglycemia [1]. In a study on rats, Na Wu infused normal saline, continuous 50% glucose, or intermittent 50% glucose for 48 hours. It was in the third group that the measured results of the influence of oxidative stress (endothelial aortic cell apoptosis and dysfunction) were most expressed [12]. It is known that oxidative stress is important in the pathogenesis of numerous neonatal diseases, such as periventricular leukomalacia, bronchopulmonary dysplasia, retinopathy of prematurity and others [13]. Only a few studies concerning glycemic variability have been carried out amongst preterm infants [13, 14, 15]. In our study the interstitial fluid glucose levels in a cohort of VLBW infants was recorded by a continuous glucose monitoring system during the first week of life. Although the CGM system produces the data needed to analyze GV, there is almost no published information on glycemic variability in preterm neonates. Only Galderisi A et al. conducted a study also based on CGM measurements in VLBW infants. It was designed to investigate whether glucose administration guided by continuous glucose monitoring is more effective than the standard monitoring approach in maintaining euglycemia in VLBW infants. Garderisi et al. demonstrated that CGM-guided glucose titration minimizes glycemic variability in preterm infants during the first week of life [14]. Tottman et al. and Fendler et al. also described glycemic variability and its impact on mortality and morbidity of VLBW infants, but GV was based on intermittent sample glucose measurements. Tottman et al. found higher glucose variability in hypoglycemic and unstable VLBW infants. It was associated with worse short term outcomes but no differences in outcomes were found at 2 years of life [3]. Fendler et al. showed also that higher glycemic variability is associated with increased early neonatal mortality among severely ill VLBW newborns [4]. To the contrary, in our cohort we did not find any association between glycemic variability and mortality before term-equivalent age. However, it is difficult to compare glycemic variability indices obtained from continuous and intermittent glucose measurement data. Different methods of calculating GV are used for these two observation methods.

In our study, the initial univariate analysis suggested an increased risk for severe ROP with hyperglycemia and glycemic variability. This is in agreement with previous studies that have shown several risk factors of ROP to be related to glucose metabolism: caloric intake, hyperglycemia, weight gain, insulin therapy, and as a consequence, IGF-1 levels [16, 17, 18, 19]. However, the multivariate logistic regression model did not show statistical significance of this association.

A possible association between IVH and hyperglycemia has been investigated for many years, but whether any relationship exists is still uncertain. On the other hand, there is no published information about the relationship of glycemic variability to IVH. In our study in the simple analysis, there was a possible association between GV and severe intraventricular hemorrhage (grade 3 and 4). However, after adjusting for important clinical covariates, we did not find this association to be significant. Additionally, no association between hyperglycemia and IVH was found.

Similarly, no association between GV and PVL could be found.

Many studies suggest an impact of GV on oxidative stress and its association with mortality and morbidity in different patient groups. There is only limited information about GV in VLBW infants. Our study shows that despite susceptibility and instability of the population, no relationship between GV and mortality and neurologic morbidities was found.

The study had limitations regarding the definition of hyperglycemia, hypoglycemia and connected real-time clinical intervention modifying interstitial fluid glucose level. Also, relatively small numbers of patients inevitably gave low power to detect differences.

## Conclusions

Further prospective studies are needed to fully investigate the impact of GV on mortality and morbidity in premature infants. The potential benefits of reducing glucose blood fluctuations in VLBW infants need to be addressed.
